# Solitary neurofibroma of the gingiva with prominent differentiation of Meissner bodies : a case report

**DOI:** 10.1186/1746-1596-5-61

**Published:** 2010-09-22

**Authors:** Jun Ohno, Teruaki Iwahashi, Ryuki Ozasa, Kazuhiko Okamura, Kunihisa Taniguchi

**Affiliations:** 1 Department of Morphological Biology, Division of Pathology, Fukuoka Dental College, Fukuoka, Japan

## Abstract

**Background:**

Oral neurofibromas are peripheral nerve sheath tumors, similar to schwannomas. Histological variations in oral neurofibromas are relatively uncommon.

**Case presentation:**

Here, we present a case of unique variation in the observed characteristics of a neurofibroma, with no relation to neurofibromatosis type-1 or von Recklinghausen disease of the skin. The neurofibroma was observed in the right mandibular gingiva of a 32-year-old Japanese woman. Histologically, it differed from conventional neurofibromas in that the tumor was composed of a mixture of fine fibrillary collagen in sheets and/or cords of neoplastic Schwann cells containing numerous clusters of Meissner bodies. Histologically, these bodies were in contact with neoplastic Schwann cells. The Meissner bodies were immunopositive for S-100 protein, neuron-specific enolase, and vimentin, but were negative for calretinin. CD34-positive spindle cells were observed around the Meissner bodies. No recurrence or signs of other tumors have been observed in the patient for 5 years after tumor resection.

**Conclusion:**

To the best of our knowledge, no formal descriptions of sporadic, solitary neurofibromas containing numerous Meissner bodies occurring in the oral cavity are available in literature. We believe that an uncommon proliferation of Meissner bodies, as seen in the present case, may result from aberrant differentiation of neoplastic Schwann cells.

## Background

Neurofibromas are benign tumors showing neural differentiation and they originate from the sympathetic, peripheral, or cranial nerves. The tumor typically presents either as a localized lesion or as part of a generalized syndrome of neurofibromatosis generally known as neurofibromatosis type-1 (NF1) or von Recklinghausen disease of the skin. NF1 is an inherited, autosomal dominant disorder characterized by multiple neurofibromas. Localized (solitary) neurofibromas most often occur as sporadic lesions in patients without NF1. In general, sporadic neurofibromas are histologically identical to those seen in NF1 [1]. They are clinically characterized by slow growth, lack of pain, and a superficial location. Histologically, these tumors are unencapsulated and comprise a mixture of Schwann cells, perineurial cells, and endoneurial fibroblasts [2-4], and are classified into major and minor variants based on their morphological features. Major variants include plexiform, diffuse, and pacinian neurofibromas, while minor variants include epithelioid, cellular, myxoid, glandular, xanthomatized, and other neurofibromas [5-7].

Although neurofibromas may often occur in the cervicofacial region, intraoral neurofibromas not related to NF-1 are relatively uncommon [8-10]. Except for plexiform and pacinian neurofibromas, limited information is available on the histological variants of oral neurofibromas. Here, we present a case of oral neurofibroma containing numerous clusters of Meissner bodies, which presented in the right mandibular gingiva of a 32-year-old Japanese woman.

## Case presentation

**Figure 1:**
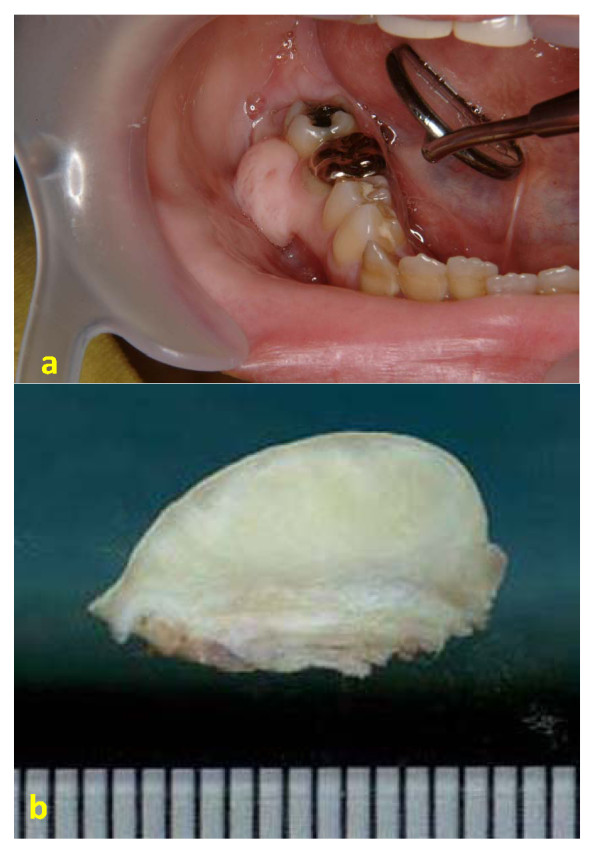
**Intraoral and gross (cut surface) findings of the tumor**. (a) Painless mass on the posterior mandible at initial presentation. (b) Cut surface showing a relatively circumscribed, yellowish-white tumor that is approximately 2.0 cm-size.

A 32-year-old Japanese woman was referred to the Oral Surgery Clinic at the Fukuoka Dental College Hospital in Fukuoka, Japan for a painless swelling on the right mandibular gingiva. She did not have a family history of neurofibromatosis and had been aware of the lesion for approximately 8 years. Intraoral examination confirmed the presence of a pendunculated swelling on the posterior mandibular facial gingiva, which was located between the first and second molars (Fig. 1a). The excised specimen measured 2.0 × 1.0 × 0.8 cm, appeared yellowish-white in color, was relatively circumscribed but unencapsulated, and showed no evidence of hemorrhage or necrosis (Fig. 1b). Detailed examination of the patient revealed no evidence of café-au-lait spots, extraperineal cutaneous neurofibromas, or other stigmata associated with von Recklinghausen disease. After tumor excision, the patient has been on regular follow-up and has shown no recurrence or complications over the last 5 years.

**Figure 2:**
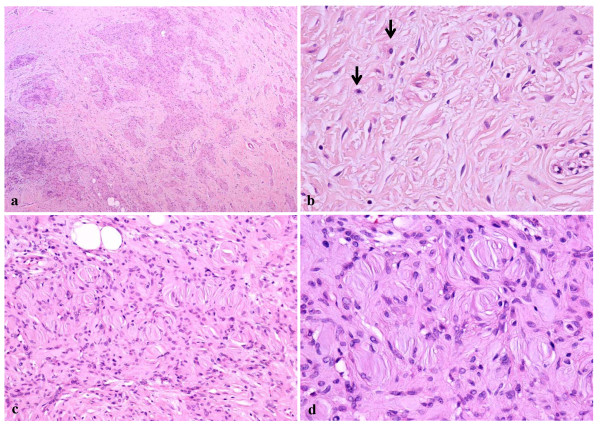
**Microscopic findings of the tumor**. (a) Fibrous and cellular components of the tumor. (b) Fibrous component of the tumor. Spindle-shaped fibroblasts and mast cells (arrows) are scattered within the fibrous connective tissue. (c) Cellular sheet of the tumor. The sheet is composed of numerous Schwann cells and Meissner bodies. (d) High magnification of the Meissner bodies. Note the lamellar structures in these bodies. (hematoxylin and eosin staining; original magnifications: a, 100 ×; b & c, 200 ×; d, 400 ×).

**Figure 3:**
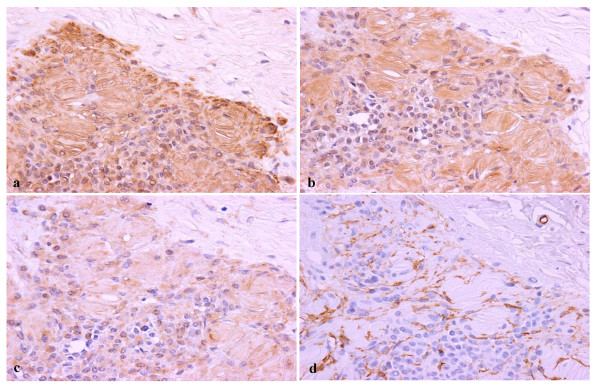
**Immunoperoxidase staining of (a) S-100 protein, (b) NSE, (c) vimentin, and (d) CD34 in the oral neurofibroma**. Lamellar structures in the Meissner bodies react intensely with anti-S-100 protein (a) and NSE (b) antibodies. Vimentin binds weakly to the Meissner bodies (c). Spindle cells around the Meissner bodies are stained with anti-CD34 antibody (d). (original magnification: a-d, 400 ×).

**Figure 4:**
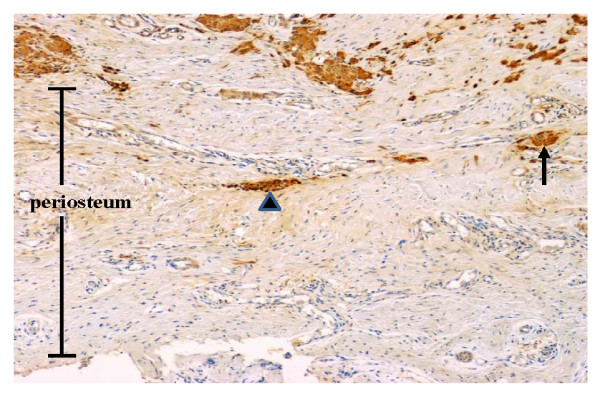
**Infiltration of S-100 protein-positive Meissner bodies into the periosteal area of the tumor**. Note the small clusters of Meissner bodies (arrow) and a nest of Schwann cells (arrow head) in the periosteal regions. (original magnification: 100 ×)

Histologically, the lesion was found to be unencapsulated and showed diffuse, circumferential infiltration of the periosteal connective tissue surrounding the neurofibroma and was surfaced with stratified squamous epithelium. The tumor was composed of paucicellular and cell-rich areas (Fig. 2a). The paucicellular areas were composed of a uniform fine fibrillary collagen matrix containing either a few spindle-shaped fibroblasts or mast cells (Fig. 2b). Prominent vascular channels were noted in these areas. The cell-rich areas displayed sheet- or cord-like growth patterns of tumor cells that had either short fusiform or rounded shapes (Fig. 2c). These cells, although histologically identical to Schwann cells, showed no evidence of cytological atypia or mitotic figures. Numerous pale eosinophilic globules containing parallel slits were observed within the cellular sheets. The globules were composed of narrow elongated cells stacked in a lamellar arrangement, and were histologically identical to the tactile corpuscle-like Meissner bodies (Fig. 2c and 2d). The nuclei of the laminar cells were located at the periphery of the corpuscles or were arranged transverse to their long axes, along the lamellae. Histologically, the Meissner bodies were in contact with the neoplastic Schwann cells. To determine the immunohistochemical characteristics of the Meissner bodies observed in the tumor, immunoperoxidase staining of the specimen was performed using the Histofine Simple Stain Kit (Nichirei, Tokyo, Japan), and the reaction products were visualized following exposure to diaminobenzidine. Antibodies to S-100 protein, calretinin, vimentin, neuron-specific enolase (NSE), and Ki-67 (MIB-1) were obtained from DakoCytomation (Kyoto, Japan). Anti-CD34 antibody was purchased from Nichirei (Tokyo, Japan). Modified microwave antigen retrieval in citrate buffer (pH 6.0) was used for analyzing antibodies to calretinin, CD34, and Ki-67. The lamellar structures of the Meissner bodies were intensely immunoreactive to both S-100 protein and NSE (Fig. 3a and 3b). The Schwann cells observed in the cellular sheets reacted to both antibodies. A few single cells in the fibrous component were stained with S-100 protein or NSE. The Meissner bodies reacted positively to vimentin but to a lesser degree compared to that for S-100 protein or NSE (Fig. 3c). CD34-positive spindle cells were found around the Meissner bodies in the cellular sheets as well as in the clusters of Meissner bodies (Fig. 3d). The vascular endothelium of the fibrous component was also positive for CD34. All cells of the fibrous and cellular components were non-reactive to the other antibodies. Small clusters of S-100 protein-positive Meissner bodies were diffusely scattered throughout the periosteum (Fig. 4). In addition, immunostaining with the nuclear proliferation marker Ki-67 (MIB-1) revealed approximately 1% positivity in the tumor cells.

## Discussion

Oral neurofibromas, like schwannoma are peripheral nerve sheath tumors. The characteristics encountered in the present case are identical to those of localized (solitary) neurofibromas not related to NF1. Furthermore, the clinical findings and Ki-67 (MIB-1) labeling index in the present case observed are similar to those of conventional neurofibromas that are often diagnosed late because they tend to grow slowly as painless nodules that produce few symptoms. In the present case, the tumor was believed to have originated from a branch of the fifth cranial nerve, similar to that seen in other cases of intraoral neurofibromas [11]. However, the present case presented with a histology that differed from conventional neurofibromas in several respects: (1) the tumor was clearly composed of paucicellular and cell-rich areas, (2) the paucicellular areas comprised a uniform matrix of fine fibrillary collagen, (3) the neoplastic Schwann cells were less elongated than those encountered in conventional neurofibromas and showed either short fusiform or rounded contours, and (4) scattered clusters of Meissner bodies were present throughout the tumor.

The most prominent finding was the scattered presence of Meissner body clusters throughout the tumor. Meissner bodies are sensitive, tactile receptors concentrated in the dermal papillae of the digital tips, palms, and soles. Similar to their location in the skin, Meissner bodies are present at the apex of the gingival connective tissue papillae within the oral mucosa and function primarily as sensitive receptors for touch stimuli [12]. Histologically, Meissner bodies are not uncommon in conventional neurofibromas [1,13,14]. However, the histological characteristics observed in the present case differed from those seen in conventional neurofibromas in that the Meissner bodies showed prominent differentiation within the tumor. Further, the lamellar structures of the Meissner bodies reacted with S-100 protein, NSE, and vimentin antibodies, which are well-established-immunohistochemical markers of Schwann cells [6]. The results presented here are similar to those from previous reports investigating immunohistochemical characteristics of Meissner bodies [13-16]. In addition to the transition of neoplastic Schwann cells to Meissner bodies, as indicated by hematoxylin and eosin staining, the immunohistochemical findings suggest that aberrant differentiation of Schwann cells and the unusual proliferation of the Meissner bodies may be related to tumor growth.

Both histological and immunohistochemical findings of the present case mimic those of diffuse neurofibromas. In general, diffuse neurofibromas are closely associated with NF1 [1] and predominantly affect the skin. The lesions are characterized by an ill-defined, diffuse infiltrations of neurofibromatous collagen fibers into the reticular dermis and subcutaneous tissue. Diffuse neurofibromas are histopathologically composed of round or slightly fusiform Schwann cells that are uniformly distributed in a delicate collagenous background and are associated with sheets of laminated bodies resembling Meissner tactile corpuscles, as seen in the present case [1,17-21]. However, this case should not necessarily be diagnosed as a diffuse neurofibroma. The degree of infiltration of the tumor in the present case was difficult to measure because the gingiva is generally composed of a relatively small amount of mucosa without abundant adipose tissue and because the tumor was located on the surface of the alveolar bone. Wagner-Meissner neurilemmoma could also be ruled out because of the absence of fibrous encapsulation and the presence of an abundant fibrous component in the tumor [16,22].

The remaining immunohistochemical results presented in this report may aid in distinguishing the tumor from schwannomas. Chaubal et al reported the presence of CD34-positive spindle cells in neurofibromas but not in schwannomas [23]. Similarly, in the present case, CD34-positive cells were found around the Meissner bodies in the tumor sheets. In addition, the tumor cells reacted negatively to calretinin antibody, which supports a recent report that suggested that positive calretinin antibody reaction occurred in almost all schwannomas but in only a small percentage of neurofibromas [24]. On the basis of the results, we infer that the present case is a unique variation of oral neurofibroma.

NF1-related neurofibromas are associated with somatic mutations at the *NF1 *gene, a tumor suppressor gene located in the pericentromeric region of chromosome 17 [1]. This gene codes for the protein neurofibromin, which regulates the *ras*-mediated cell growth pathway [25,26]. Recent studies have reported that mutations at the *NF1 *gene in Schwann cells are responsible for tumorigenesis of NF1-associated neurofibromas [27,28]. It has been suggested that the *NF1 *mutations produce abnormal neurofibromin, leading to increased levels of activating proteins (e.g., p21^ras ^and p13) [29], which contribute to the cellular proliferation of Schwann cells associated with neurofibromas. A production of abnormal neurofibromin may also be related to the incidence of malignant peripheral nerve sheath tumor arising in neurofibromas [30]. In contrast, the pathogenesis of sporadic neurofibromas remains inadequately investigated. Using G-banding and fluorescence *in situ *hybridization analyses, Storlazzi et al demonstrated that somatic inactivation of the *NF1 *gene occurred through chromosomal translocations in sporadic neurofibromas, thereby suggesting the importance of *NF1 *inactivation in the tumorigenesis of sporadic neurofibromas [31]. This finding leads us to speculate that Schwann cells exhibiting an inactivated *NF1 *gene may lead to the development of a unique neurofibroma, as the one presented in this report.

## Conclusion

An uncommon case of a sporadic, solitary oral neurofibroma with prominent Meissner bodies was identified. No formal description of a similar case was found in scientific literature. Both histological and immunohistochemical findings in this case suggest that the aberrant differentiation of neoplastic Schwann cells may be implicated in Meissner body clusters, which are uncommon in a solitary neurofibroma.

## Consent

Written informed consent was obtained from the patient for publication of this case report and accompanying images. A copy of the written consent is available for review by the Editor-in-Chief of *Diagnostic Pathology*.

## Competing interests

The authors declare that they have no competing interests.

## Authors' contributions

JO performed the histological examination, analyzed the case, and prepared the manuscript. TI and RO participated in the immunostaining. KO and KT participated in conducting pathological examinations. All authors have read and approved the final manuscript.
